# Improvement Effect of Lotus Leaf Flavonoids on Carbon Tetrachloride-Induced Liver Injury in Mice

**DOI:** 10.3390/biomedicines8020041

**Published:** 2020-02-24

**Authors:** Tongji Liu, Fang Tan, Xingyao Long, Yanni Pan, Jianfei Mu, Xianrong Zhou, Runkun Yi, Xin Zhao

**Affiliations:** 1 Chongqing Collaborative Innovation Center for Functional Food, Chongqing University of Education, Chongqing 400067, China; liutj@foods.ac.cn (T.L.); longyaoyao@foods.ac.cn (X.L.); panyanni@foods.ac.cn (Y.P.); mujianfei@foods.ac.cn (J.M.); zhouxr@foods.ac.cn (X.Z.); 2 Chongqing Engineering Research Center of Functional Food, Chongqing University of Education, Chongqing 400067, China; 3 Chongqing Engineering Laboratory for Research and Development of Functional Food, Chongqing University of Education, Chongqing 400067, China; 4 College of Biological and Chemical Engineering, Chongqing University of Education, Chongqing 400067, China; 5 Department of Public Health, Our Lady of Fatima University, Valenzuela 838, Philippines; tanfang@foods.ac.cn

**Keywords:** carbon tetrachloride, liver injury, lotus leaf flavonoids, mRNA, mice

## Abstract

In this study, the effect of lotus leaf flavonoids (LLF) on carbon tetrachloride (CCl_4_)-induced liver injury in mice was studied. CCl_4_ was injected intraperitoneally to induce liver injury in Kunming mice. Mice were treated with LLF by gavage, and the mRNA expression levels in serum and liver were detected. Compared with the model group, LLF significantly reduced the liver index and serum aspartate aminotransferase (AST), alanine aminotransferase (ALT), triglyceride (TG), and total cholesterol (TC) levels in mice with CCl_4_-induced liver injury. Pathological observation showed that LLF effectively reduced morphological incompleteness and hepatocyte necrosis in CCl_4_-treated liver tissue. The result of quantitative polymerase chain reaction (qPCR) indicated that LLF significantly up-regulated the mRNA expression levels of copper/zinc superoxide dismutase (Cu/Zn-SOD), manganese superoxide dismutase (Mn-SOD), and catalase (CAT) and down- regulated the expression levels of tumor necrosis factor-alpha (TNF-α), nuclear factor kappa B (NF-κB), and interleukin-1β (IL-1β) (*p* < 0.05). Thus, LLF is an active ingredient that ameliorates liver injury, and it has good application prospect.

## 1. Introduction

The liver is an important organ in metabolism and physiology, and liver injury causes serious harm to the body. Multiple factors can cause liver injury; chemical-induced liver injury is a common cause of liver dysfunction, and severe chemical-induced liver injury can even cause liver cirrhosis and liver cancer [1]. When carbon tetrachloride enters the animal body, it can directly enter the hepatocyte, and dissolve the lipid of mitochondrial membrane, thus it affects the structure and function of mitochondria, reduces the synthesis of enzyme protein, and causes the destruction and release of enzymes, thus affecting the generation of metabolism and energy, and making the hepatocyte degenerate and necrotic. Using this animal model, we can observe the metabolic disorders of sugar, fat, protein and pigment, the decrease of liver detoxification function, and the morphological changes of malnutrition, fatty degeneration and liver necrosis [2]. Carbon tetrachloride (CCl_4_) is a commonly used chemical inducer of liver injury in the laboratory. During the process of inducing liver injury, CCl_4_ causes the secretion of many inflammatory factors by the hepatocytes, thus aggravating the inflammatory effect and liver injury. At the same time, CCl_4_ causes lipid peroxidation of liver microsomes, leads to lipid peroxidation of the cell membrane, destroys the cell membrane, and induces liver injury [3]. Other chemicals can also cause serious damage to the liver. Clinical research also showed that paracetamol could cause liver damage to both children and adults. In serious cases, it would also lead to acute liver failure and life-threatening states [4,5].

Lotus leaves are dry leaves of the *Nelumbo nucifera* Gaertn, which is a well known traditional Chinese medicine and has functions such as clearing away the heat and dampness, preventing hair loss and promoting hair growth, cooling the blood, and hemostasis [6,7]. Modern medical studies have revealed that the main active components of lotus leaves are flavonoids and alkaloids [8,9]. Currently, the flavonoids in lotus leaves have been reported to reduce the blood lipid level, to have anti-allergy, anti-cancer, anti-aging, bacteriostatic, and anti-oxidation functions, and to be helpful in treating cardiovascular diseases [10,11]. The results showed that flavonoids and alkaloids in lotus leaf were the main active components that promoted lipid metabolism and played the role of lipid-lowering [12]. The research also showed that lotus leaf contained flavonoids and the lotus leaf had the functions of anti-atherosclerosis, protecting vascular endothelium and regulating arrhythmia [13]. At the same time, the research showed that free radicals could be removed by flavonoids of lotus leaf, and inoleic acid oxidation could be inhibited. Lotus leaf is a non-toxic and safe antioxidant [14]. Due to their high medicinal value and low cost, they have become a research hotspot.

In this study, CCl_4_ was used for treating the liver to establish a mouse model of chemical-induced liver injury. The effect of lotus leaf flavonoids (LLF) on the prevention of CCl_4_^-^induced liver injury in mice was observed, and related indices in serum and the liver tissue of mice were measured by molecular biological methods. On the basis of these findings, the effects of lotus leaf flavonoids on liver injury in mice were elucidated. The results will be useful in the development and utilization of lotus leaf flavonoids, and will provide a theoretical basis for their application in drug processing and research and the development of health products.

## 2. Experimental Section

### 2.1. Materials and Instruments

Lotus leaf: Anhui Xile Garden Food Co., Ltd., Anqing, Anhui, China; FL-3 macroporous resin: Hefei Sifeng Biotechnology Co., Ltd., Hefei, Anhui, China; aspartate aminotransferase (AST), alanine aminotransferase (ALT), triglyceride (TG), total cholesterol (TC) kits: Nanjing Jiancheng Bioengineering Institute, Nanjing, Jiangsu, China; Trizol: Invitrogen, Carlsbad, CA, USA; oligo Primer DT, RNase inhibitor, dNTP mix, RevertAid RT, Master Mix, primer, ddH_2_O: Thermo Fisher Scientific, Inc., Waltham, MA, USA; dimethyl sulfoxide, silymarin (positive control drug): ≥ 95%, Sigma, St. Louis, MO, USA; 95% ethanol, analytical pure ethanol, methanol, glacial acetic acid, acetonitrile, chloroform, isopropanol: Shanghai Shiyi Chemical Reagent Co., Ltd., Shanghai, China, FL-3 macroporous resin: Jiangsu Hecheng New Material Co., Ltd., Nanjing, Jiangsu, China; CCl_4_: Tianjin Zhiyuan Chemical Reagent Co., Ltd., Tianjin, China. In total, 70% ethanol was diluted by 95% ethanol and distilled water, 0.1% glacial acetic acid was diluted by glacial acetic acid and distilled water, and 50% ethanol was diluted by analytical pure ethanol and distilled water. Kaempferitrin, hyperoside, astragalin, phloridzin, quercetin standard products: Shanghai Yuanye Biotechnology Co., Ltd., Shanghai, China.

Kunming mice: Chongqing Medical University, Chongqing, China.

Ultimate 3000 high performance liquid chromatography, Dionex ultimate 3000 DAD detector, Stepone Plus qPCR instrument: Thermo Fisher Scientific, Inc., Waltham, MA, USA; BX43 optical microscope: Olympus, Tokyo, Japan.

SPSS22.0 software: IBM, New York, NY, USA.

### 2.2. Extraction of Lotus Leaf Flavonoids (LLF)

Firstly, 1000 g dry lotus leaves were weighed and added with 70% ethanol (*v*/*v*) in a ratio of 1:20 (lotus leaves to ethanol, *w*/*w*) after grounding in a beaker. Then, the beaker was sealed with a plastic film and placed in a water bath. The temperature of the water bath was maintained at 60 °C for 3 h, and then the beaker was taken out and cooled. The mixture was filtered to obtain a crude extract of LLF, followed by slow loading into a glass column with FL-3 macroporous resin. The filtrate that passed through the resin firstly was discarded. The column was eluted with 70% ethanol until the resin became colorless. The eluted solution was collected in a beaker, and ethanol was removed from the solution by rotary evaporation to obtain the LLF extract.

### 2.3. Composition Analysis of Lotus Leaf Flavonoids (LLF)

The components of LLF were analyzed by HPLC-DAD (quaternary gradient pump, DAD detector), the liquid chromatography conditions were C18 column (Agilent zorbaxsb-c, 5 μm, 4.6 × 250 mm), mobile phase A: 0.1% glacial acetic acid, mobile phase B: 100% acetonitrile, column temperature: 35 °C, flow rate: 0.5 mL/min, detection wavelength: 360 nm, injection volume: 10 μL, chromatographic running time: 40 min. The gradient elution conditions of the mobile phase are shown in Table 1.

### 2.4. Animal Experiment

LLF was prepared into 50 and 100 mg/kg LLF crude extract b.w. (body weight of mice) of solution with distilled water according to mouse body weight, and silymarin was prepared into 100 mg/kg b.w. of solution with distilled water also according to mouse body weight. Fifty Kunming mice (six weeks old, male) were randomly assigned into the following five groups: normal group, model group, silymarin group (100 mg/kg b.w.), low-dose LLF group (50 mg/kg b.w.), and high-dose LLF group (100 mg/kg b.w.), and 10 mice in each group. The mice were maintained in a controlled facility at temperature 25 ± 2 °C in relative humidity 50 ± 5% with a 12 h light/dark cycle and free access to a standard mouse diet and water. After being acclimated for one week before the experiment, each mouse in the normal group and the model group was treated with 0.2 mL normal saline per day by oral gastric lavage. Silymarin and LLF were diluted with distilled water and stored in a refrigerator at −20 °C. Each mouse in the high- and low-dose LLF groups was treated with 0.2 mL LLF (50 and 100 mg/kg b.w.) at the corresponding concentration per day by oral gastric lavage. Each mouse in the silymarin group was treated with 0.2 mL silymarin solution per day at a dose of 100 mg/kg. The experiment lasted for 14 days. All mice were allowed free access to water and standard feed. On the 14th day, all mice, except those in the normal group, were injected intraperitoneally with a chemical inducer CCl_4_ (0.8%; CCl_4_: peanut oil = 1:125, *v*/*v*). After injection, all mice were fasted, but access to free drinking water was allowed. After fasting for 24 h, blood was collected from the eyeballs, the neck was cut off, and the liver was resected for use [15]. The weight of the liver was measured, and liver index was determined according to the following equation: liver index = (liver weight/body weight) × 100. This study was approved by the Ethics Committee of Chongqing Collaborative Innovation Center for Functional Food (201906005B) (25 June 2019), Chongqing, China.

### 2.5. Measurement of Serum Indices

In the next stage, 0.5 mL blood was centrifuged at 4 °C for 10 min to obtain the serum, and it was stored at −80 °C for use for one week. Serum levels of AST, ALT, TG, and TC were measured in triplicate by experimental kits.

### 2.6. Observation of Liver Histopathology

Briefly, 1/2 of the right lobe of the liver was fixed in 10% formalin, dehydrated with 95% ethanol, and then replaced with xylene to extract ethanol in liver tissue. After being embedded in paraffin, the sections were cooled and stained by hematoxylin-eosin (H and E), and observed under an optical microscope.

### 2.7. Measurement of mRNA Expression in the Liver by Quantitative Polymerase Chain Reaction (Q-PCR)

Firstly, 100 mg of liver tissue was homogenized with 1 mL Trizol and 200 μL chloroform by a homogenizer. The supernatant was collected after centrifugation at 4 °C for 15 min at 14,000 rpm/min, and then it was added with isopropanol in a ratio of supernatant: isopropanol = 1:1 (*v*/*v*), placed at 4 °C for 15 min, and then centrifuged at 4 °C at 14,000 rpm/min for 20 min to obtain RNA. RNA was diluted to 1 μg/μL with enzyme free water; 9 μL oligo Primer DT was mixed with 1 μL diluted RNA, and then the mixture was placed in a PCR machine to complete a thermal cycle at 65°C for 5 min; further, 4 μL 5 × reaction buffer, 1 μL ribolock RNase inhibitor, 2 μL 100 mM dNTP mix, and 1 μL RevertAid RT were added, and then the mixture was placed in the PCR machine at 42°C for 60 min and 70 °C for 5 min to generate cDNA; 1 μL cDNA, 10 μL Master Mix, 2 μL primer, and 7 μL ddH_2_O were mixed, and placed in the qPCR instrument, using the following program: 94 °C for 30 s, 40 cycles, 58 °C for 30 s, and 72 °C for 50 s. Finally, the cycle threshold (CT) value was measured at 75 °C for 10 min, using the equation 2^-ΔΔCT^ for calculation [16]. The primer sequence is shown in Table 2.

### 2.8. Measurement of Serum Indices

All experiments were performed in triplicate, and the results were averaged. SPSS22.0 software was used to analyze the data. One-way analysis of variance (one way ANOVA) was used to analyze the significant difference at a level of *p* < 0.05.

## 3. Results

### 3.1. Composition of Lotus Leaf Flavonoids (LLF)

**Figure 1:**
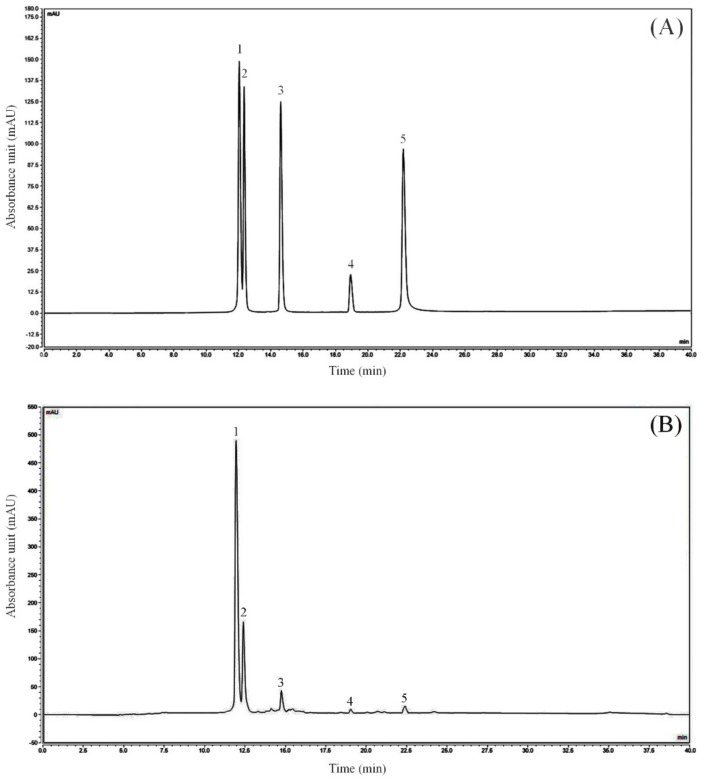
Flavonoids constituents of lotus leaves. (**A**) Standard chromatograms; (**B**) Lotus leaves flavonoids chromatograms. 1: kaempferitrin; 2: hyperoside; 3: astragalin; 4: phloridzin; 5: quercetin.

The kaempferitrin, hyperoside, astragalin, phloridzin, and quercetin standards purchased were prepared into 1 mg/mL standard solution with 50% methanol solution. 50 μL standard solutions were respectively transferred into 5 mL EP tube, and then filtered with 0.22 μm filter membrane. After the prepared sample and standard solution were filtered, 0.5 mL of these solutions were injected into the injection vial through a micro syringe for three parallel detections. By changing the injection quantity of the mixed standard, the sample was injected as 2, 4, 6, 10, 12 and 14 μL, with the injection quantity as the abscissa and the peak area as the ordinate, six chromatograms were obtained, and the scatter plot was drawn to calculate the linear regression curve and the linear regression coefficient of each standard (Table 3, Figure 1). The peak area of kaempferitrin, hypericin, astragalin, phlorizin, and quercetin could be obtained by HPLC-DAD. The injection volume of the mixed standard was 2 μL, while that of LLF was 10 μL. The quantitative limit was 0.6–3.0 mg/L, and the detection limit was 0.2–1.0 mg/L. By calculating the peak area, the content of each component in the LLF could be calculated. The peak area was shown in Table 4, and the calculation formula was as follows: M_(LLF)_ = C_(standard)_×V_(standard)_×A_(LLF)_×LLF dilution ratio/A_(standard)_, M_(LLF)_: LLF weight (mg); C_(standard)_: standard concentration (mg/mL); V_(standard)_: standard injection volume (mL); A_(LLF)_: LLF peak area; A_(standard)_: standard peak area. The result of the HPLC-DAD analysis showed that the flavonoids contained in lotus leaves included kaempferitrin, hypericin, astragalin, phlorizin, and quercetin (Table 5). The peak areas of kaempferitrin and hypericin were the largest in LLF, which indicates that these two flavonoids are the main components of lotus leaves.

### 3.2. Measurement of the Liver Index

As shown in Table 6, the liver weight and liver index in the model group were the highest among the five groups. Compared with the normal group, the liver weight and liver index in the model group were significantly higher (*p* < 0.05), indicating successful modeling. The liver weight and liver index in the normal group were the lowest, and the liver weight and liver index in the silymarin group and the LLF groups were reduced to different extents compared with those in the model group. The liver weight and liver index in the high-dose LLF group were closer to those in the silymarin group than the low-dose LLF group, indicating that LLF can effectively ameliorate CCl_4_-induced liver injury.

### 3.3. Observation of Liver Histopathology

**Figure 2:**
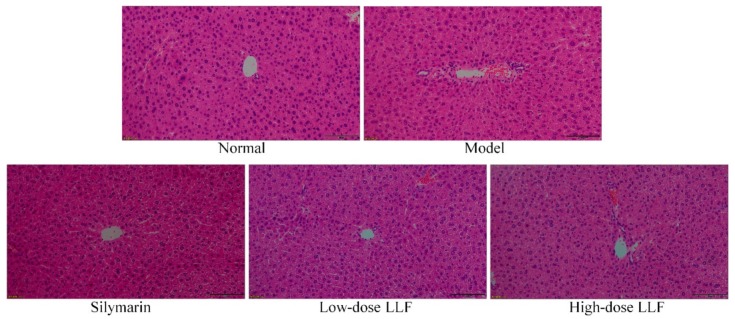
Hematoxylin and eosin (H and E) pathological observations of hepatic tissues in mice. Magnification 100×. Silymarin: mice treated with silymarin (100 mg/kg); low-dose LLF: mice treated with a low concentration of lotus leaf flavonoids (50 mg/kg); high-dose LLF: mice treated with a high concentration of lotus leaf flavonoids (100 mg/kg).

Histopathological section is an important technique to detect liver injury. Through observation and analysis of the liver tissue section, liver injury can be evaluated. As shown in Figure 2, the HE stained section of the liver illustrated that the morphology and structure of normal hepatocytes were normal, complete, and arranged in an orderly manner. In the model group, the liver tissue showed a large area of necrotic cells, infiltration of inflammatory cells, and destruction and uneven arrangement of the cell structure. Compared with the model group, LLF and silymarin reduced the necrosis of hepatocytes caused by CCl_4_ to a certain extent and induced a decrease in the incomplete morphology of hepatocytes, in which the effect of high-dose LLF was more significant, thus indicating that the protective effect of high-dose LLF on liver injury is better and is similar to the effect of silymarin.

### 3.4. Measurement of Serum Indices

As shown in Table 7, after CCl_4_-induced liver injury, serum levels of AST, ALT, TG, and TC in the model group were significantly higher than those in the normal group (*p* < 0.05). The corresponding serum levels in the LLF group were significantly lower than those in the model group (*p* < 0.05), but they were higher than those in the silymarin group. Moreover, the corresponding serum levels in the high-dose LLF group were significantly lower than those in the low-dose LLF group.

### 3.5. Expression Levels of Copper/Zinc Superoxide Dismutase (Cu/Zn-SOD), Manganese Superoxide Dismutase (Mn-SOD), and Catalase (CAT) in Liver Tissues

**Figure 3:**
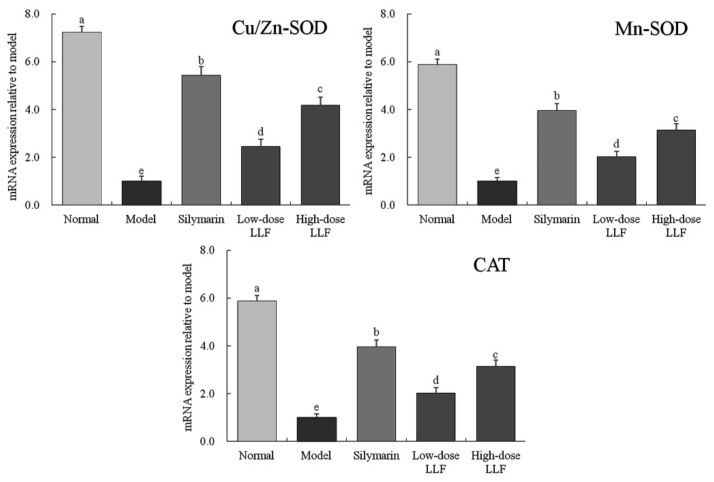
*Cu/Zn-SOD*, *Mn-SOD*, and *CAT* mRNA expression in liver tissues of the mice. Sample data in each group came from a normal distribution. ^a–e^ Mean values with different letters in the bar are significantly different (*p* < 0.05) according to Tukey’s honestly significant different test. Silymarin: mice treated with silymarin (100 mg/kg); low-dose LLF: mice treated with a low concentration of lotus leaf flavonoids (50 mg/kg); high-dose LLF: mice treated with a high concentration of lotus leaf flavonoids (100 mg/kg).

As illustrated in Figure 3, the expression levels of *Cu/Zn-SOD*, *Mn-SOD*, and *CAT* in the liver tissues of normal mice were higher than those in the liver tissues of other mice (*p* < 0.05). In the model group, the expression levels of *Mn-SOD*, *Cu/Zn-SOD*, and *IκB-α* were decreased, and LLF significantly alleviated these decreases (*p* < 0.05). Moreover, the effect of high-dose LLF was more significant and it was close to that of silymarin.

### 3.6. Expression Levels of Tumor Necrosis Factor-Alpha (TNF-α), Nuclear Factor Kappa B (NF-κB), and Interleukin-1β (IL-1β) in Liver Tissues

**Figure 4:**
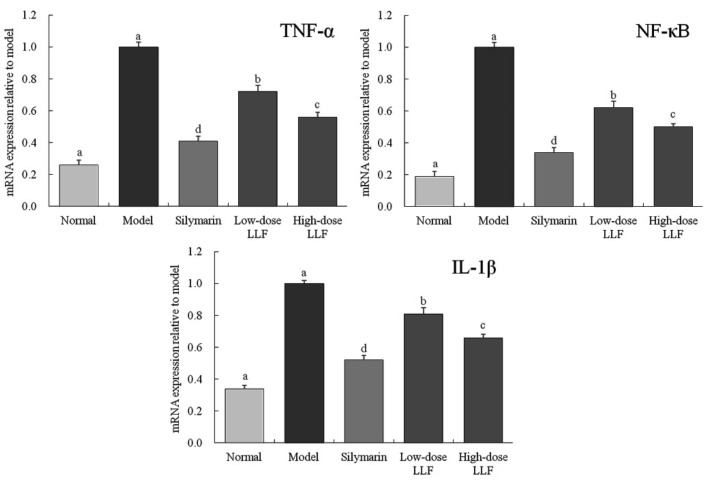
*TNF-α*, *NF-κB*, and *IL-1β* mRNA expression in liver tissues of the mice. Sample data in each group came from a normal distribution. ^a–e^ Mean values with different letters in the bar are significantly different (*p* < 0.05) according to Tukey’s honestly significant different test. Silymarin: mice treated with silymarin (100 mg/kg); low-dose LLF: mice treated with a low concentration of lotus leaf flavonoids (50 mg/kg); high-dose LLF: mice treated with a high concentration of lotus leaf flavonoids (100 mg/kg).

As illustrated in Figure 4, compared with the normal group, the expression levels of *TNF-α*, *NF-κB*, and *IL-1β* in the liver tissues of the model group were significantly enhanced by CCl_4_ (*p* < 0.05). Compared with the model group, LLF and silymarin down-regulated the expression levels of *TNF-α*, *NF-κB*, and *IL-1β* enhanced by CCl_4_, and the corresponding expression levels in the high-dose LLF group were significantly lower than those in the low-dose LLF group, but they were slightly higher than those in the normal group and the silymarin group. Thus, LLF could significantly down-regulate the expression levels of *TNF-α*, *NF-κB*, and *IL-1β* in mice with liver injury (*p* < 0.05), and the effect was close to that of silymarin.

## 4. Discussion

Silymarin can protect the liver cell membrane, recover the liver function quickly, and prevent the liver injury caused by many kinds of liver poisons, especially chemical liver injury, such as alcoholic liver injury. Silymarin could play the role of auxiliary protective function on these injuries. It has the special effect of relieving hangovers, promoting the synthesis of liver sugar, and inhibiting alcohol liver injury [19]. Therefore, in this study, silymarin was used as a positive drug control, and the effect of the LLF was observed by comparing with the positive drug of silymarin.

The liver is the largest internal organ in the abdominal cavity, and liver index has been widely used to evaluate CCl_4_-induced liver injury, such as evaluating the effect of *Ganoderma lucidum* polysaccharides, grape seed proanthocyanidins and *Bidens pilosa* Linnaeus (*Bidens pilosa* L.) flavonoids [20,21,22]. High liver index is one of the manifestations of liver injury [23]. This study has also shown that LLF can reduce the liver index of mice with liver injury, the reducing ability of high-dose LLF was significantly higher than that of low-dose LLF (*p* < 0.05), and the effect is similar to that of silymarin.

ALT and AST are two transaminase markers for detecting liver injury. After the hepatocytes are damaged, AST, ALT, and other enzymes are released into the blood [24]. Different levels of AST and ALT can be detected in the serum of mice corresponding to different degrees of liver injury. Therefore, the activities of ALT and AST in serum can accurately reflect the degree of liver injury [25]. CCl_4_-induced liver injury leads to the transfer of fatty acids to the liver tissue and increased TG content in the liver. At the same time, TC is also an indicator of lipid peroxidation caused by liver injury. After liver injury, TG and TC levels increase due to lipid peroxidation [26,27]. It could be seen that LFF could effectively regulate these liver function indexes and protect the liver, and the effects of high-dose LLF were better than those of low-dose LLF (*p* < 0.05).

Mn-SOD and Cu/Zn-SOD are isomers of SOD in the body. They are two types of SOD radical scavengers with different ions as active centers. High-level Mn-SOD and Cu/Zn SOD can effectively inhibit the toxic effect caused by O_2_^-^ and can protect the liver tissue [28]. The activities of Mn-SOD and Cu/Zn-SOD are decreased after liver injury. By observing the expression levels of Mn-SOD and Cu/Zn-SOD in the tissues, we can evaluate the degree to which the body is affected by oxidation reaction [29]. CAT, an important antioxidant enzyme in the body, can inhibit oxidative stress, reduce oxidation of CCl_4_ in the body, and inhibit liver injury by eliminating H_2_O_2_ in the body [30]. It could be seen from the experimental results that LLF had a good role in regulating oxidative emergency, which could effectively avoid oxidative damage of liver tissue caused by carbon tetrachloride, thus reducing liver damage. Meanwhile, the Mn-SOD, Cu/Zn-SOD and CAT levels’ up-regulation abilities of high-dose LLF were stronger than those of low-dose LLF (*p* < 0.05).

TNF-α is the most critical pro-inflammatory cytokine, which plays a positive feedback role in the activation of NF-κB [31]. It is not only the activator of NF-κB, but also the product of its activation. Low concentration of TNF-α can regulate the growth, differentiation, and regeneration of hepatocytes, but high concentration TNF-α does not only induce hepatocyte apoptosis, but it is also the main factor causing hepatocyte damage [32]. NF-κB is an important transcription factor in vivo, which regulates the gene expression required for an inflammatory response, such as liver injury [33]. High NF-κB level indicates liver injury [34]. IL-1β is an inflammatory cytokine secreted by Kupffer cells in the liver, and it attracts neutrophils and causes the release of inflammatory mediators, thus aggravating liver injury [35]. It could be seen from the experimental results that LLF could reduce the inflammatory response and liver tissue damage, and high-dose LLF showed stronger reducing effects than low-dose LLF (*p* < 0.05).

Kaempferitrin, hypericin, astragalin, phlorizin, and quercetin are active compounds with higher anti-inflammatory and anti-oxidation effects [36,37,38,39,40]. The improvement effect of LLF on CCl_4_-induced liver injury also arises from the effect of these five chemicals, especially the synergistic effect.

Lotus and its leaves have certain biological activities, in particular, some studies showed that they had good antioxidant activity, and their antioxidant activity also played a protective role in cells, especially on liver cells and liver [41,42,43,44]. In addition, the lotus seed was also rich in alkaloids, which could inhibit liver injury [1]. These studies showed that the stems, leaves and fruits of lotus could have a positive role in protecting the liver. This study also showed that the polyphenols contained in lotus leaves had a good intervention in alcoholic liver injury.

## 5. Conclusions

The results of this study showed that LLF can improve liver injury induced by CCl_4_ in mice. LLF can alleviate the liver tissue lesion induced by CCl_4_ and can restore serum indices in mice with liver injury to normal levels. The qPCR experiment further confirmed that LLF effectively restores the expression related to oxidation and inflammation in the liver tissues of mice with liver injury close to the normal level. LLF has a good preventive effect on liver injury induced by CCl_4_ in mice, and it has a similar effect to silymarin. Therefore, LLF has a good development and utilization potential. LFF could be used in the production of traditional Chinese medicine, so that it can be used as a new drug.

## Figures and Tables

**Table 1:** Gradient elution conditions of mobile phase.

Time (min)	Current Speed (mL/min)	%A	%B
−10.000	0.500	88.0	12.0
0.000	0.500	88.0	12.0
30.000	0.500	60.0	40.0
35.000	0.500	0.0	100.0
40.000	0.500	0.0	100.0

**Table 2:** Sequences of the primers used for the mice liver tissue.

Gene Name	Sequence
*Cu/Zn–SOD* [17]	Forward: 5′–AACCAGTTGTGTTGTCAGGAC–3′
	Reverse: 5′–CCACCATGTTTCTTAGAGTGAGG–3′
*Mn–SOD* [17]	Forward: 5’-CAGACCTGCCTTACGACTATGG-3’
	Reverse: 5’-CTCGGTGGCGTTGAGATTGTT-3’
*CAT* [17]	Forward: 5’-GGAGGCGGGAACCCAATAG-3’
	Reverse: 5’-GTGTGCCATCTCGTCAGTGAA-3’
*TNF-α* [17]	Forward: 5’-GACCCTCAGACTCAGATCATCCTTCT-3’
	Reverse: 5’-ACGCTGGCTCAGCCACTC-3’
*NF-κB* [18]	Forward: 5′-GAGGCACGAGGCTCCTTTTCT-3′
	Reverse: 5′-GTAGCTGCATGGAGACTCGAACA-3′
*IL-1β* [17]	Forward: 5’-CTCCATGAGCTTTGTACAAGG-3’
	Reverse: 5’-TGCTGATGTACCAGTTGGGG-3’
*GAPDH* [17]	Forward: 5’-AGGTCGGTGTGAACGGATTTG-3’
	Reverse: 5’-GGGGTCGTTGATGGCAACA-3’

*Cu/Zn–SOD*: cuprozinc–superoxide dismutase; *Mn–SOD*: manganese superoxide dismutase; *CAT*: catalase; *TNF-α*: tumor necrosis factor alpha; *NF-κB*: nuclear factor kappa-B; *IL-1β*: Interleukin-1 beta; *GAPDH*: glyceraldehyde-3-phosphate dehydrogenase.

**Table 3:** Linear regression equation of standard product.

Standard	Linear Regression Equation	R^2^
Kaempferitrin	y = 8.1347 × + 3.7415	0.9844
Hyperoside	y = 3.1862 × + 9.5029	0.9897
Astragalin	y = 11.338 × + 0.8515	0.9994
Phloridzin	y = 0.9844 × + 0.3039	0.9843
Quercetin	y = 10.386 ×−3.0995	0.9990

**Table 4:** Composition of lotus leaf flavonoids.

Chemical Compound	A_(standard)_ (mAU)	A_(LLF)_ (mAU)
Kaempferitrin	18.8283 ± 0.0003	139.6697 ± 0.0001
Hyperoside	13.4423 ± 0.0004	32.7726 ± 0.0003
Astragalin	23.0850 ± 0.0003	1.1600 ± 0.0002
Phloridzin	2.2368 ± 0.0002	0.0452 ± 0.0003
Quercetin	17.7730 ± 0.0003	0.4064 ± 0.0003

**Table 5:** Composition of lotus leaf flavonoids.

Compound	Kaempferitrin	Hyperoside	Astragalin	Phloridzin	Quercetin
Content (mg/g)	397.85 ± 0.17	195.60 ± 0.15	10.34 ± 0.13	0.41 ± 0.03	4.73 ± 0.11

**Table 6:** The body weight, liver weight, and liver index of mice (*n* = 10).

Group	Body Weight (g)	Liver Weight (g)	Liver Index
Normal	35.87 ± 1.36 ^a^	1.24 ± 0.16 ^c^	3.46 ± 0.26 ^d^
Model	35.14 ± 2.82 ^a^	2.41 ± 0.45 ^a^	6.86 ± 0.44 ^a^
Silymarin	35.51 ± 2.11 ^a^	1.58 ± 0.31 ^bc^	4.44 ± 0.27 ^c^
Low-dose LLF	35.66 ± 2.19 ^a^	2.10 ± 0.26 ^a^	5.89 ± 0.31 ^b^
High-dose LLF	35.91 ± 2.28 ^a^	1.71 ± 0.21 ^b^	4.76 ± 0.16 ^c^

Values are mean ± standard deviation (*n* = 10/group). Sample data in each group came from a normal distribution. ^a–^^d^ Mean values with different letters over the same column are significantly different (*p* < 0.05) according to Tukey’s honestly significant different test. Silymarin: mice treated with silymarin (100 mg/kg); low-dose LLF: mice treated with a low concentration of lotus leaf flavonoids (50 mg/kg); high-dose LLF: mice treated with a high concentration of lotus leaf flavonoids (100 mg/kg). ALT, alanine aminotransferase; AST, aspartate aminotransferase; TG, triglycerides; TC, total cholesterol.

**Table 7:** Levels of AST, ALT, TG, and TC in mice serum (*n* = 10).

Group	ALT (U/L)	AST (U/L)	TG (mmol/L)	TC (mmol/L)
Normal	12.05 ± 1.17 ^e^	10.83 ± 0.55 ^e^	0.36 ± 0.06 ^e^	1.51 ± 0.21 ^e^
Model	63.88 ± 4.10 ^a^	55.17 ± 3.28 ^a^	2.01 ± 0.16 ^a^	5.92 ± 0.42 ^a^
Silymarin	28.76 ± 2.06 ^d^	19.69 ± 2.13 ^d^	0.63 ± 0.06 ^d^	2.29 ± 0.28 ^d^
Low-dose LLF	47.82 ± 2.11 ^b^	40.55 ± 1.97 ^b^	1.35 ± 0.07 ^b^	4.03 ± 0.22 ^b^
High-dose LLF	34.18 ± 2.23 ^c^	26.82 ± 1.48 ^c^	0.86 ± 0.05 ^c^	3.14 ± 0.20 ^c^

Values are mean ± standard deviation (n = 10/group). Sample data in each group came from a normal distribution. ^a–^^e^ Mean values with different letters over the same column are significantly different (p < 0.05) according to Tukey’s honestly significant different test. Silymarin: mice treated with silymarin (100 mg/kg); low-dose LLF: mice treated with a low concentration of lotus leaf flavonoids (50 mg/kg); high-dose LLF: mice treated with a high concentration of lotus leaf flavonoids (100 mg/kg). ALT, alanine aminotransferase; AST, aspartate aminotransferase; TG, triglycerides; TC, total cholesterol.
